# Prevalence, characteristics, and plasmid dynamics of *mcr-1* positive Enterobacteriaceae in Hainan, China: a preliminary genomic investigation

**DOI:** 10.3389/fmicb.2025.1689159

**Published:** 2026-01-14

**Authors:** Suimei Wang, Xiaosheng Han, Yan Sheng, Wang Zhou, Hui Huang, Xiaobin Wei

**Affiliations:** Haikou Affiliated Hospital of Central South Uniersity Xiangya School of Medicine, Haikou, China

**Keywords:** colistin resistance, Enterobacteriaceae, horizontal gene transfer, IncI2, *mcr-1*

## Abstract

**Introduction:**

The global spread of the plasmid-mediated colistin resistance gene *mcr-1* poses a serious threat to public health. This study aimed to conduct a preliminary characterization of the epidemiology and genomic features of Enterobacteriaceae carrying the *mcr-1* gene in a hospital setting in Hainan, China.

**Methods:**

A total of 2,700 *Enterobacteriaceae* strains, including 2,200 fecal samples and 500 respiratory, blood, and urine isolates, were collected from Haikou People’s Hospital between October 2020 to September 2024. Specifically, the *mcr-1* gene was screened by PCR. Antimicrobial susceptibility testing was performed with the VITEK 2 system. Four *mcr-1* positive strains underwent whole-genome sequencing using Illumina and Nanopore platforms, which were combined with CARD, multilocus sequence typing (MLST), and plasmid analysis to elucidate resistance mechanisms.

**Results:**

The positivity rate for *mcr-1* was 0.15% (4/2,700). All positive isolates were identified as *Escherichia coli*, with two strains originating from urine and two from fecal samples. Antimicrobial susceptibility testing showed that the urine isolates (C29 and C180) were extensively drug resistant (XDR). The fecal strain S321.4 was multidrug resistant (MDR), while S118.1 was sensitive. Patients with XDR/MDR strains had recent antibiotic exposure and invasive procedures. Whole-genome analysis revealed that MLST types of the strains were diverse (ST410, ST167, ST11165, ST1266), and *mcr-1* was located on plasmids of IncI2 or IncX4 types. The IncI2 plasmid carried a complete conjugative operon. Plasmid C180_5 harbored *bla*_CTX-M-199_ through IS150, forming a multidrug resistance plasmid. Strain C29 exhibited a reduced colistin minimum inhibitory concentration (MIC) of 0.5 μg/mL due to disruption of *mcr-1* by IS3, which likely impairs gene function. However, this requires further functional validation.

**Conclusion:**

This preliminary study indicates a low prevalence of *mcr-1* in our setting. However, the genomic identification of conjugative plasmids, including one carrying both *mcr-1* and an extended-spectrum β-lactamase gene, highlights a tangible risk for horizontal co-transfer of resistance. The association of these isolates with healthcare exposures underscores the need for ongoing surveillance to monitor plasmid evolution in hospital ecosystems.

## 1 Introduction

Antibiotic resistance represents has become a major global public health challenge. In particular, the issue of antibiotic resistance in Gram-negative bacteria has become increasingly severe (Mancuso et al., 2023). Colistin is the last line of defense for treating multidrug-resistant Gram-negative bacterial infections, and its emerging resistance has attracted widespread attention (Nang et al., 2021; Jiang et al., 2020). The mobile colistin resistance gene (*mcr*), first discovered in 2015, represents a new resistance mechanism. This gene has been found in various bacteria, and its transmission capability and potential threat to public health cannot be ignored (Al Mana et al., 2022; Vu Thi Ngoc et al., 2022). The current research status on the mcr gene indicates that, although past studies have mainly focused on screening of the *mcr-1* gene, the emergence of other mcr family genes such as *mcr-2* to *mcr-10* in recent years has also drawn researchers’ attention (Zhang et al., 2025; Ewers et al., 2022). Studies have shown that bacteria carrying the *mcr-1* gene are increasingly widespread globally, especially in the intestinal microbiota of animals and humans (Huang et al., 2023; Mei et al., 2024). The rapid global dissemination of *mcr-1* is primarily mediated by its location on mobile genetic elements, especially plasmids, rather than by clonal expansion of bacterial strains (Kim et al., 2022). Plasmids of incompatibility groups *IncI2*, *IncX4*, and *IncHI2* have been identified as predominant vectors for *mcr-1* (Liu et al., 2016). Critically, many of these are conjugative plasmids that carry a complete set of genes encoding a type IV secretion system (T4SS), which enables the plasmid to transfer a copy of itself to a recipient bacterium through conjugation (Sun et al., 2018). This process allows for efficient horizontal spread of *mcr-1* across diverse bacterial populations in the gut, environment, and clinical settings. Furthermore, the genetic context of *mcr-1* is often flanked by insertion sequences (IS) (Dominguez et al., 2025). These IS elements facilitate mobilization and capture of *mcr-1* into various plasmid backbones and can promote co-localization of *mcr-1* with other antibiotic resistance genes (e.g., extended-spectrum beta-lactamase or carbapenemase genes), leading to emergence of multidrug-resistant plasmids that can defy last-resort treatment options (Li et al., 2017). The colonization of multidrug-resistant bacteria in the gastrointestinal tract may increase the risk of bacterial translocation and infections, which is also the main reason for the prevalence of the colistin resistance gene *mcr-1* in *Escherichia coli* (Karim et al., 2023). Resistance to colistins in intestinal bacteria is considered to have particularly important clinical significance. *Escherichia coli* is the main species carrying *mcr-1* and is also a major pathogen causing diarrhea (Johura et al., 2020). Research has shown that the detection rate of *mcr-1* carrying *Escherichia coli* in clinical samples has been increasing year by year, especially among children, indicating that this gene is of significant importance in clinical infections (Xie et al., 2022).

To address a critical knowledge gap regarding this region, we characterized the epidemiology and genomic features of *mcr-1* harboring Enterobacteriaceae in Hainan, China, a region with unique ecological and demographic features. We collected isolates from hospitalized patients at Haikou People’s Hospital (October 2020 to September 2024) and utilized whole-genome sequencing to explore resistance gene profiles and plasmid contexts of *mcr-1* positive *Escherichia coli*. As the first in-depth investigation in this region, our work not only provides insights into local resistance mechanisms but also establishes a essential baseline for ongoing surveillance and future research into the plasmid transmission dynamics of *mcr-1*.

## 2 Materials and methods

### 2.1 Source of the strain

Fecal samples from 2,200 hospitalized patients at Haikou People’s Hospital were collected from October 2020 to September 2024, with each patient providing only one sample. Additionally, 500 unique strains of *Enterobacteriaceae* were isolated from respiratory, blood, and urine specimens of hospitalized patients. The fecal samples were inoculated onto China blue agar plates and incubated at 35 °C for 18–24 h; presumptive *Enterobacteriaceae* colonies were then selected and transferred to blood agar plates for further culturing. Meanwhile, the *Enterobacteriaceae* isolates from the respiratory, blood, and urine specimens were revived and inoculated onto blood agar plates and incubated at 35 °C for 18–24 h for subsequent use.

### 2.2 Identification of microbial strains and detection of *mcr-1*

Identification was performed using the MALDI Biotyper system (Bruker, Germany) with matrix-assisted laser desorption/ionization time-of-flight mass spectrometry (MALDI-TOF MS). According to the manufacturer’s instructions, bacterial cells cultured on specific media at 37 °C for 18 to 24 h were evenly applied onto the target plate. Subsequently, 1 μL of *α*-cyano-4-hydroxycinnamic acid (CHCA) matrix solution was added to each spot; after allowing it to air dry at room temperature, mass spectrometry analysis was conducted.

#### 2.2.1 Detection of *mcr-1*

Polymerase chain reaction (PCR) was performed using *mcr-1*-specific primers, with reference to the literature (Liu et al., 2016). All 2,700 isolated strains were diluted in sterile water and then heated in a metal heating block for 10 min. After centrifugation, the supernatant was used as the template to detect PCR products by 1% agarose gel electrophoresis. The PCR products from samples suspected of carrying the *mcr-1* gene were purified and sequenced at Shanghai Shenggong Biological Engineering Co., Ltd. The obtained sequences were compared for sequence similarity using the NCBI database^1^ and the EzBioCloud database.^2^

### 2.3 Antimicrobial susceptibility testing

Antimicrobial susceptibility testing was performed on the experimental bacterial strains using the Vitek 2 Compact system with GN334 and GN335 cards. The drugs tested included colistin, cefoperazone-sulbactam, piperacillin-tazobactam, ceftazidime, cefepime, aztreonam, imipenem, meropenem, tobramycin, amikacin, levofloxacin, ciprofloxacin, tigecycline, and doxycycline, following the manufacturer’s instructions. Susceptibility results were interpreted according to CLSI M100 standards for *Enterobacteriaceae*. *Escherichia coli* ATCC 25922 was used as a quality control strain in the antimicrobial susceptibility testing.

### 2.4 Whole-genome sequencing and bioinformatics analysis

We transported four strains of *Enterobacteriaceae* carrying the *mcr-1* gene to Guangdong Meige Gene Technology Co., Ltd. using dry ice for whole genome sequencing. The sequencing utilized a combination of technologies from the Illumina NovaSeq X Plus and Nanopore PromethION platforms. Subsequently, we assembled sequencing reads from both Illumina and Nanopore platforms using Unicycler.^3^ The average sequencing depth of Illumina sequencing was 450X. The N50 values of the C180, C29, S118.1, and S321.4 genome assemblies were 4,838,437, 4,725,936, 4,649,392, and 4,949,069. Next, we used the Resistance Gene Identifier (RGI) software tool from the Comprehensive Antibiotic Resistance Database (CARD)^4^ to identify antibiotic resistance genes in the genomic data of these four strains. We typed the strains using the MLST scheme and submitted the assembled contig files to the PubMLST BIGSdb platform to identify the MLST sequence types of the strains based on seven housekeeping genes of *Escherichia coli* (adk, fumC, gyrB, icd, mdh, purA, recA). Furthermore, we performed serotyping using the ECTyper v2.0.0 tool. We identified plasmid replication factor types using PlasmidFinder v2.0.1 and analyzed mobile genetic elements (MGEs) using ISfinder. Integrative conjugative elements (ICE) were identified using ICEberg v3.0. We generated circular plasmid maps and comparisons using Geneious^5^ and clinker v0.0.31. Plasmid compatibility was evaluated using Plascad v1.17. All bioinformatics tools use default parameters. The whole genome sequences of four strains carrying the *mcr-1* gene C29, C180, S321.4, and S118.1 have been deposited in the GenBank.

## 3 Results

### 3.1 Clinical cases of bacterial strains carrying the *mcr-1* gene

Among 2,700 Enterobacteriaceae isolates screened, four (0.15%) were confirmed to carry the *mcr-1* gene, all identified as *Escherichia coli*. Two strains (C29, C180) were isolated from urine samples of patients with urinary tract infection and severe pneumonia, respectively. The other two strains (S321.4, S118.1) were from fecal samples of patients admitted for an ovarian cyst and angina pectoris, respectively (Table 1). A notable pattern emerged from the clinical metadata (Table 1). Three of the four patients (C29, C180, S321.4) had undergone invasive procedures (e.g., ureteroscopic lithotripsy, tracheostomy, total hysterectomy) and had received antibiotic therapy (piperacillin/sulbactam or amoxicillin/clavulanate) either prior to or during hospitalization. None of the patients received colistin during their hospital stay.

**Table 1:** Clinical characteristics of patients harboring *mcr-1* positive *Escherichia coli*.

Isolate	C29	C180	S321.4	S118.1
Age	57	87	49	62
Sex	F	F	F	F
Source	Urine	Urine	Feces	Feces
Department	Urology	Respiratory ICU	Gynecology	Cardiology
Diagnosis	Urinary tract infection	Severe pneumonia	Ovarian cyst	Angina pectoris
Invasive procedure	Ureteroscopic lithotripsy	Tracheostomy	Total hysterectomy	None
Antibiotics used	Piperacillin/sulbactam	Piperacillin/sulbactam	Amoxicillin/clavulanate	None
Whether colistin was used during hospitalization	None	None	None	None

### 3.2 Antimicrobial susceptibility testing

Susceptibility results are detailed in Table 2. The two urine isolates (C29, C180) were XDR. The fecal isolate S321.4 was MDR, while S118.1 was susceptible to most agents. Strain C29 exhibited a colistin MIC of ≤0.5 μg/mL, whereas the other three had MICs of 2 μg/mL.

**Table 2:** Antibiotic susceptibility testing of four *Escherichia coli* strains with the *mcr-1* gene.

Isolate	MIC (μg/mL)													
	COL	CSL	TZP	CAZ	FEP	ATM	IPM	MEM	TOB	AMK	LEV	CIP	TGC	DOX
C29	≤0.5	≥64	≥128	≥32	≥16	≤1	≥16	≥16	≥16	≥64	≥8	≥4	≤0.5	≥16
C180	2	≥64	≥128	≥32	≥32	≥64	≥16	≥16	≥16	≤2	≥8	≥4	≤0.5	≥16
S321.4	2	≤8	8	≥32	≥32	≥32	≤0.25	≤0.25	≥8	≤2	≥8	≥4	≤0.5	≥16
S118.1	2	≤8	≤4	0.5	≤0.12	≤1	≤0.25	≤0.25	≤1	≤2	≤0.12	≤0.25	≤0.5	≥16

COL, colistin; CSL, cefoperazone-sulbactam; TZP, piperacillin-tazobactam; CAZ, ceftazidime; FEP, cefepime; ATM, aztreonam; IPM, imipenem; MEM, meropenem; TOB, tobramycin; AMK, amikacin; LEV, levofloxacin; CIP, ciprofloxacin; TGC, tigecycline; DOX, doxycycline.

### 3.3 Genomic features

The whole-genome sequences of four bacterial strains were obtained through a combination of short-read sequencing (Illumina platform) and long-read sequencing (Nanopore). This approach resulted in the assembly of a single chromosome and multiple circular plasmids for each strain. The whole-genome sequencing data showed that the *mcr-1* gene in the four *Escherichia coli* isolates was located on plasmids (specific plasmid types are listed in Table 3). MLST results indicated that the strains belong to sequence types ST410, ST167, ST11165, and ST1266, respectively, and that they are genetically distinct. Various antimicrobial resistance genes were identified on the chromosomes and plasmids, including *acrD*, *AAC(3)-IVa*, *emrA*, *emrB*, *emrR*, *mdtH*, *bla*_OXA-1_, *bla*_TEM-1_, *bla*_NDM-5_. More details are provided in Table 3.

**Table 3:** Genomic features of four strains of *Escherichia coli* that carry the *mcr-1* gene.

Genomic features		C29	C180	S321.4	S118.1
Chromosome/plasmids		1 + 6	1 + 11	1 + 3	1 + 7
MLST		ST410	ST167	ST1266	ST11165
Chromosome/plasmid		-:H9	O101:H9	O188:H34	O100/O154:H25
*mcr-1* plasmid		IncX4	IncI2	IncI2	IncI2
Resistance genes	Colistin	*mcr-1, PmrF, eptA*	*mcr-1, PmrF, eptA*	*mcr-1, PmrF, eptA*	*mcr-1, PmrF, eptA*
	Aminoglycosides	*AcrD, kdpE, AAC(6′)-Ib10, AAC(3)-IVa, APH(4)-Ia*	*AcrD, aadA2, aadA, kdpE, AAC(3)-IVa, APH(4)-Ia, APH(6)-Id, APH(3″)-Ib*	*KdpE, acrD, AAC(3)-IId, aadA22, APH(6)-Id, APH(3′)-Ia*	*AcrD, kdpE, aadA2, APH(6)-Id, APH(3″)-Ib, aadA*
	Fluoroquinolones	*EmrA, emrB, emrR, mdtH*	*EmrA, emrB, emrR, mdtH, QnrS1*	*MdtH, gyrA, emrR, emrA, emrB, parC, QnrS1*	*EmrB, emrA, emrR, mdtH*
	β-lactams	*bla*_EC-15_, *bla*_OXA-1_, *bla*_TEM-1_, *bla*_NDM-5_	*bla*_EC-15_, *bla*_NDM-5_, *bla*_OXA-10_, *bla*_TEM-1_, *bla*_CTX-M-199_	*bla*_EC-13_, *bla*_CTX-M-55_	*bla*_EC-14_, *bla*_TEM-1_
	Sulfonamides	*sul1, sul2*	*sul3*	*sul2, sul3*	*sul1, sul2, sul3*
	Tetracycline	*EmrK, emrY, tet(A)*	*emrK, emrY, tet(A)*	*emrK, emrY, tet(A), tet(M)*	*emrK, emrY, tet(A), tet(M)*
	Fosfomycin	*GlpT, mdtG*	*mdtG*	*mdtG, mdtG*	*GlpT, mdtG*
	Rifamycin	*arr-3*	*arr-2*	*arr-2*	

### 3.4 Analysis of four *Escherichia coli* strains that carry plasmids with the *mcr-1* gene

**Figure 1:**
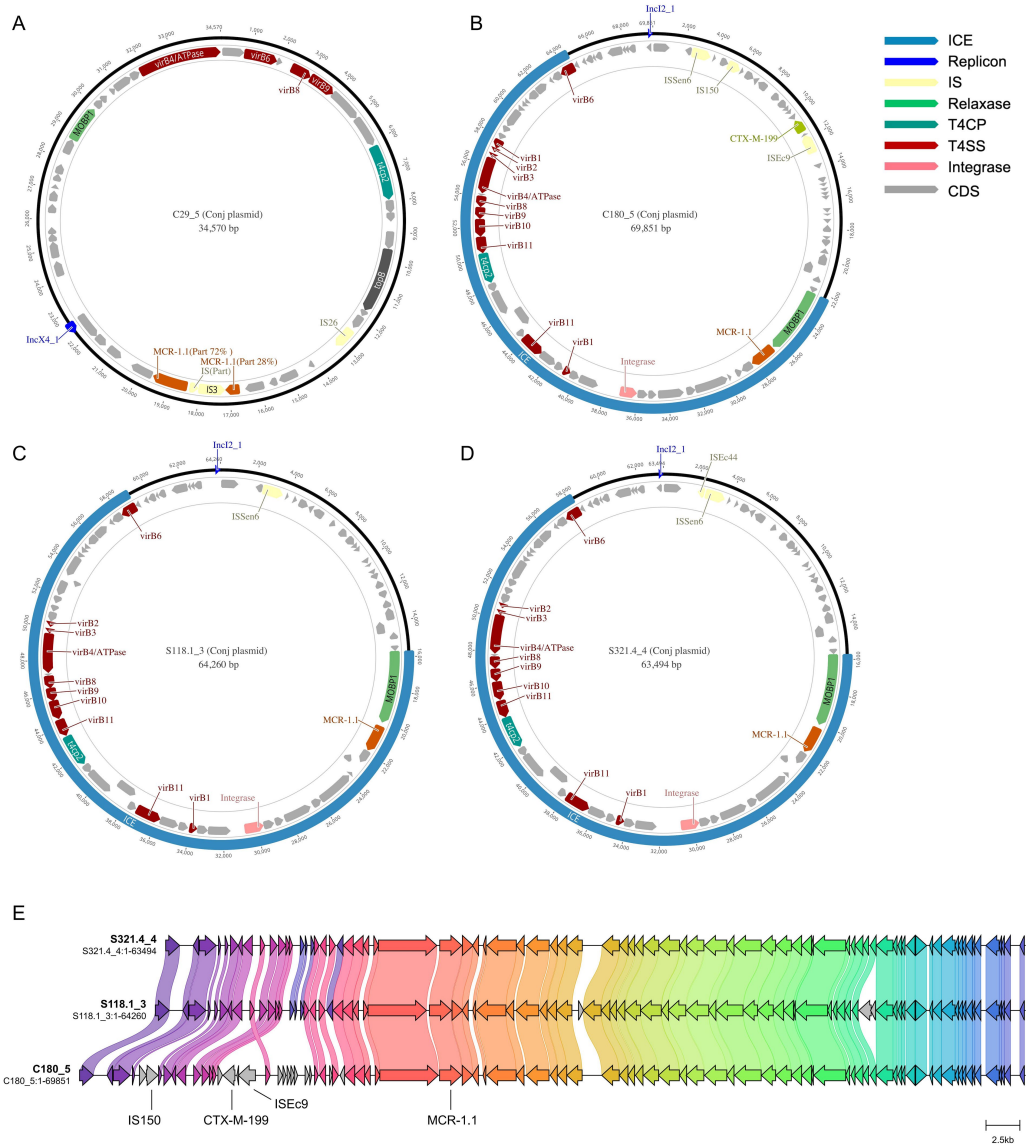
Circular sequence alignment of plasmids from four *Escherichia coli* strains **(A–D)** carrying the *mcr-1* gene, and comparison of plasmid skeleton diagrams of *Escherichia coli* strains **(A–C)** carrying the *mcr-1* gene **(E)**.

**Figure 2:**
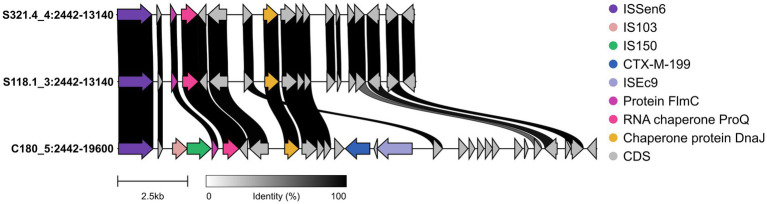
Genetic context of *bla*_CTX-M-199_ and proposed IS150-mediated capture event.

In this study, the plasmids C180_5, S118.1_3, and S321.4_4 that carry the *mcr-1* gene all belong to IncI2-type conjugative plasmids and have a complete conjugative transfer operon (virB1–virB11). Bioinformatic analysis predicted that these plasmids possess the genetic backbone for conjugative transfer, as they carry a complete set of T4SS genes (virB1–virB11). Among them, the C180_5 plasmid has the most insertion sequences (IS), including ISEc9, IS150, and ISSen6, indicating a strong potential for horizontal gene transfer and potentially accelerating the recombination of resistance genes, as shown in Figures 1A–D. The S118.1_3 and S321.4_4 plasmids contain the insertion sequence (IS) ISEc44, which might enhance the plasmids’ evolutionary potential. Additionally, the C29_5 plasmid belongs to the IncX4 type conjugative plasmid, in which the *mcr-1* gene is split into two parts due to recombination with the insertion sequence (IS) IS3, constituting 72 and 28% of the full *mcr-1* gene, respectively, and its conjugative transfer operon is incomplete. Since the plasmid backbones are highly similar, C180_5, S118.1_3, and S321.4_4 are of the same plasmid type. A linear comparison of the plasmids shows that C180_5 has an extra 2.8 kb region compared to the other two, which includes IS150 and *bla*_CTX-M-199_. Therefore, *bla*_CTX-M-199_ might be captured and integrated into this plasmid through transfer mediated by the nearby IS150 from other plasmids. More details can be found in Figure 1E. The linear mapping results of the plasmid region indicated that, compared with the other two plasmids, the C180_5 plasmid had gene fragment insertions in the regions of 4,420–5,790 bp and 10,214–17,614 bp. Among them, the insertion sequence ISEc9 was detected at 253 bp downstream of the *bla*_CTX-M-199_ gene. Insertion sequences IS150 and IS103 were detected at 4,803 bp upstream. Based on the above sequence characteristics, we speculate that the process of *bla*_CTX-M-199_ gene insertion into the C180_5 plasmid might be directly mediated by the adjacent insertion sequence ISEc9 downstream (the two are the closest and have the highest mediation probability). It can also be mediated by the composite turntable structure formed by the synergy of ISEc9, IS150 and IS103. More details can be found in Figure 2.

## 4 Discussion

Antibiotic resistance has become a major challenge in global public health, particularly in the treatment of infections caused by multidrug-resistant Gram-negative bacteria, making drug selection increasingly complicated. Furthermore, the emergence of the *mcr-1* gene, which mediates colistin resistance, creates a serious challenge for clinical treatment (Zhang et al., 2018; Che et al., 2023). The *mcr-1* gene has been found not only in animal-derived bacteria but has also gradually spread and colonized the human gut, posing a significant threat to traditional clinical treatment methods. In this context, the emergence of Gram-negative bacteria carrying the *mcr-1* gene in the gut could lead to endogenous infections. Therefore, it is crucial to monitor the spread of these bacteria, especially those carrying the *mcr-1* gene, as this will help develop effective infection control strategies to protect public health. This study conducted genomic analysis on *Escherichia coli* carrying *mcr-1* in a tertiary hospital in Hainan, China. Although the observed prevalence was very low (0.15%), in-depth analysis of the four positive isolates revealed some notable mechanisms and clinically relevant findings.

The low detection rate aligns with some recent reports from China (Mei et al., 2024). However, the clinical context of the carriers is notable. Three of the four patients underwent invasive procedures (e.g., tracheostomy, hysterectomy) and received broad-spectrum antibiotics (Table 1). This aligns with established risk factors for healthcare-associated colonization and infection with multidrug-resistant organisms (Garcia-Parejo et al., 2025). It suggests that even in low-prevalence settings, healthcare ecosystems can act as focal points for the selection and potential transmission of such resistant strains.

In antimicrobial susceptibility testing, strains C29 and C180, which were derived from urine samples, showed resistance to several antibiotics. These strains also carried different types of resistance genes. Furthermore, these findings align with the results of the antimicrobial susceptibility testing, confirming that these strains are XDR. In contrast, strain S321.4 from fecal samples is MDR, while S118.1 is sensitive to most antibiotics. This might be linked to their history of antibiotic exposure during hospitalization because C29, C180, and S321.4 all underwent invasive surgeries and were given antimicrobial drugs during their hospital stay (Wang et al., 2017). The presence of the *mcr-1* gene, along with other resistance genes like *bla*_OXA-1_, *bla*_TEM-1_, and *bla*_NDM-5_, may therefore lead to treatment failures by limiting effective therapeutic options and complicate infection control efforts by facilitating the spread of resistant strains. We need to strengthen monitoring and research on resistance genes in antibiotic use and infection control strategies to lessen the threat that resistant strains pose to public health.

In this study, all *mcr-1* genes were found on plasmids, and the strains had different MLST sequence types (ST410, ST167, ST11165, ST1266). This aligns with the global pattern of the gene being primarily transferred horizontally via plasmids. It is not typically disseminated through clonal dissemination (Kim et al., 2022; Li et al., 2022). This mode of transmission significantly increases the likelihood of *mcr-1* spreading in bacterial populations. Furthermore, analysis showed that the *mcr-1*-carrying plasmids from C180_5, S118.1_3, and S321.4_4 are all of the IncI2 type. These plasmids contain a complete T4SS gene cluster and possess efficient conjugative transfer capabilities, potentially facilitating further spread of resistance genes (Sun et al., 2023). As shown in Figure 1E, the plasmids from C180_5, S118.1_3, and S321.4_4 exhibit highly similar backbone structures and replicon types (IncI2), suggesting these plasmids might spread through conjugative transfer, which requires experimental verification.

To clear this study plasmid global positioning and potential sources, we will assemble a complete plasmid sequences with JGI/IMG^6^ than a database. The results showed that the IncX4 plasmid C29_5 was completely consistent with IMGPR_plasmid_2923339551_000002 at the nucleotide level. The latter is an IncX4 plasmid known to be globally transmissible and carrying the complete *mcr-1* gene. On the other hand, the skeleton structures of the three IncI2 plasmids (C180_5, S118.1_3, S321.4_4) are highly similar to IMGPR_plasmid_2909303794_000002. However, on the basis of this common skeleton, C180_5 inserted an exogenous region of approximately 2.8 kb through IS150, which contained the*bla*_CTX-M-199_ gene, a feature that did not exist in IMGPR_plasmid_2909303794_000002 and its highly similar plasmids. The proximity of *bla*_CTX-M-199_ to IS150 indicates recent mobilization. This co-localization has direct public health implications. The use of broad-spectrum cephalosporins or penicillins to treat infections can co-select this plasmid, thereby maintaining and transmitting *mcr-1* even without colistin selection pressure, a well-documented phenomenon in other drug-resistant gene combinations (Wang et al., 2017).

Another intriguing observation was the IS3-mediated disruption of the *mcr-1* gene in strain C29, which coincides with a susceptible colistin MIC, presents a fascinating finding. While this could represent a stochastic insertion event, it also invites speculation about a potential fitness cost associated with maintaining a functional *mcr-1* gene in the absence of colistin selective pressure. It is plausible that the constitutive expression of *mcr-1* phosphatidylethanolamine transferase might burden cellular resources or membrane integrity, disadvantaging the host bacterium in a competitive environment where colistin is not used. If this inactivation reflects a selective advantage in the hospital setting (devoid of colistin pressure), it could temporarily lower phenotypic resistance rates while allowing a crippled resistance gene to persist. Notably, such an interrupted gene remains a latent reservoir; the *mcr-1* fragments could potentially regain functionality through precise excision of the IS3 element or recombination events, posing a hidden threat. This hypothesis warrants future investigation through competitive growth assays and genetic complementation studies.

This study has limitations. Its single-center design and small number of positive isolates preclude broad epidemiological conclusions. The findings are descriptive and hypothesis-generating. However, the value lies in the detailed genomic “early warning” it provides.

## 5 Conclusion and recommendations

In conclusion, this initial investigation found a low prevalence of *mcr-1* in our hospital. However, the genomic characterization revealed clear risks: the presence of *mcr-1* on conjugative plasmids, including one co-carrying a clinically relevant ESBL gene, demonstrates an efficient pathway for horizontal resistance gene spread. The association with typical healthcare exposures further highlights the hospital setting as a crucial arena for monitoring and intervention. Continuous surveillance integrating genomic and epidemiological data is essential to track the evolution of these resistant plasmids and inform effective infection control strategies in Hainan and similar regions.

## Data Availability

The genome sequences generated in this study are available in the NCBI GenBank database under accession numbers JBPZSL000000000, JBPZSM000000000, JBPZSN000000000, and JBPZSO000000000.

## Footnotes

1
https://blast.ncbi.nlm.nih.gov/Blast.cgi

2
https://www.ezbiocloud.net/

3
https://github.com/rrwick/Unicycler

4
https://card.mcmaster.ca

5
https://www.geneious.com

6
https://genome.jgi.doe.gov/portal/IMG_PR/IMG_PR.home.html

## References

[ref1] Al Mana H. Johar A. A. Kassem I. I. Eltai N. O. (2022). Transmissibility and persistence of the plasmid-borne mobile colistin resistance gene, *mcr-1*, harbored in poultry-associated *E. coli*. Antibiotics 11:774. doi: 10.3390/antibiotics11060774, 35740180 PMC9220209

[ref2] Che Y. Wu R. Li H. Wang L. Wu X. Chen Q. . (2023). Characterization of two novel colistin resistance gene *mcr-1* variants originated from *Moraxella* spp. Front. Microbiol. 14:1153740. doi: 10.3389/fmicb.2023.1153740, 37260682 PMC10228737

[ref3] Dominguez J. E. Martino F. Lovera R. Casanova N. A. Seah C. Cavia R. . (2025). Genomic characterization of plasmids of *mcr-1*-positive *Escherichia coli* isolated from cohabiting rats, dairy cattle and pigs. BMC Vet. Res. 21:271. doi: 10.1186/s12917-025-04665-4, 40229806 PMC11995477

[ref4] Ewers C. Göpel L. Prenger-Berninghoff E. Semmler T. Kerner K. Bauerfeind R. (2022). Occurrence of *mcr-1* and *mcr-2* colistin resistance genes in porcine *Escherichia coli* isolates (2010–2020) and genomic characterization of *mcr-2*-positive *E. coli*. Front. Microbiol. 13:1076315. doi: 10.3389/fmicb.2022.1076315, 36569100 PMC9780603

[ref5] Garcia-Parejo Y. Gonzalez-Rubio J. Garcia Guerrero J. Gomez-Juarez Sango A. Cantero Escribano J. M. Najera A. (2025). Risk factors for colonisation by multidrug-resistant bacteria in critical care units. Intensive Crit. Care Nurs. 86:103760. doi: 10.1016/j.iccn.2024.103760 38987037

[ref6] Huang Y. Wang Z. Liu Z. Huan Q. Liu Y. Li R. . (2023). Gigantol restores the sensitivity of mcr carrying multidrug-resistant bacteria to colistin. Phytomedicine 117:154886. doi: 10.1016/j.phymed.2023.154886, 37269755

[ref7] Jiang Y. Zhang Y. Lu J. Wang Q. Cui Y. Wang Y. . (2020). Clinical relevance and plasmid dynamics of *mcr-1*-positive *Escherichia coli* in China: a multicentre case-control and molecular epidemiological study. Lancet Microbe 1, e24–e33. doi: 10.1016/S2666-5247(20)30001-X, 35538906

[ref8] Johura F. T. Tasnim J. Barman I. Biswas S. R. Jubyda F. T. Sultana M. . (2020). Colistin-resistant *Escherichia coli* carrying *mcr-1* in food, water, hand rinse, and healthy human gut in Bangladesh. Gut Pathog. 12:5. doi: 10.1186/s13099-020-0345-2, 32002025 PMC6986151

[ref9] Karim M. R. Zakaria Z. Hassan L. Faiz N. M. Ahmad N. I. (2023). The occurrence and molecular detection of *mcr-1* and *mcr-5* genes in Enterobacteriaceae isolated from poultry and poultry meats in Malaysia. Front. Microbiol. 14:1208314. doi: 10.3389/fmicb.2023 37601372 PMC10435970

[ref10] Kim Y. J. Seo K. H. Kim S. Bae S. (2022). Phylogenetic comparison and characterization of an *mcr-1*-harboring complete plasmid genome isolated from Enterobacteriaceae. Microb. Drug Resist. 28, 492–497. doi: 10.1089/mdr.2021.0164, 35180355 PMC9058865

[ref11] Li W. Bai X. Sheng H. Chen J. Wang Z. Wang T. . (2022). Conjugative transfer of *mcr-1*-bearing plasmid from Salmonella to *Escherichia coli in vitro* on chicken meat and in mouse gut. Food Res. Int. 157:111263. doi: 10.1016/j.foodres.2022.111263, 35761575

[ref12] Li R. Xie M. Zhang J. Yang Z. Liu L. Liu X. . (2017). Genetic characterization of *mcr-1*-bearing plasmids to depict molecular mechanisms underlying dissemination of the colistin resistance determinant. J. Antimicrob. Chemother. 72, 393–401. doi: 10.1093/jac/dkw411, 28073961

[ref13] Liu Y. Y. Wang Y. Walsh T. R. Yi L. X. Zhang R. Spencer J. . (2016). Emergence of plasmid-mediated colistin resistance mechanism MCR-1 in animals and human beings in China: a microbiological and molecular biological study. Lancet Infect. Dis. 16, 161–168. doi: 10.1016/S1473-3099(15)00424-7 26603172

[ref14] Mancuso G. De Gaetano S. Midiri A. Zummo S. Biondo C. (2023). The challenge of overcoming antibiotic resistance in carbapenem-resistant gram-negative bacteria: “Attack on Titan”. Microorganisms 11:1912. doi: 10.3390/microorganisms11081912, 37630472 PMC10456941

[ref15] Mei C. Y. Jiang Y. Ma Q. C. Lu M. J. Wu H. Wang Z. Y. . (2024). Low prevalence of *mcr-1* in *Escherichia coli* from food-producing animals and food products in China. BMC Vet. Res. 20:40. doi: 10.1186/s12917-024-03891-6, 38297289 PMC10832210

[ref16] Nang S. C. Azad M. A. K. Velkov T. Zhou Q. T. Li J. (2021). Rescuing the last-line polymyxins: achievements and challenges. Pharmacol. Rev. 73, 679–728. doi: 10.1124/pharmrev.120.000020, 33627412 PMC7911091

[ref17] Sun J. Li X. P. Yang S. S. Yang Y. Yin Z. Y. Li S. M. . (2018). Co-selection of *mcr-1* and other antibiotic resistance genes by non-colistin antibiotics in *Escherichia coli*. Front. Microbiol. 9:2551. doi: 10.3389/fmicb.2018.02551, 30416494 PMC6212470

[ref18] Sun X. Zhang L. Meng J. Peng K. Huang W. Lei G. . (2023). The characteristics of *mcr*-bearing plasmids in clinical *Salmonella enterica* in Sichuan, China, 2014 to 2017. Front. Cell. Infect. Microbiol. 13:1240580. doi: 10.3389/fcimb.2023.1240580, 37705933 PMC10495832

[ref19] Vu Thi Ngoc B. Le Viet T. Nguyen Thi Tuyet M. Nguyen Thi Hong T. Nguyen Thi Ngoc D. Le Van D. . (2022). Characterization of genetic elements carrying *mcr-1* gene in *Escherichia coli* from the community and hospital settings in Vietnam. Microbiol. Spectr. 10:e0135621. doi: 10.1128/spectrum.01356-2, 35138158 PMC8826730

[ref20] Wang Y. Tian G. B. Zhang R. Shen Y. Tyrrell J. M. Huang X. . (2017). Prevalence, risk factors, outcomes, and molecular epidemiology of *mcr-1*-positive Enterobacteriaceae in patients and healthy adults from China: an epidemiological and clinical study. Lancet Infect. Dis. 17, 390–399. doi: 10.1016/S1473-3099(16)30527-8, 28139431

[ref21] Xie J. Liang B. Xu X. Yang L. Li H. Li P. . (2022). Identification of *mcr-1*-positive multidrug-resistant *Escherichia coli* isolates from clinical samples in Shanghai, China. J. Glob. Antimicrob. Resist. 29, 88–96. doi: 10.1016/j.jgar.2022.02.008, 35182776

[ref22] Zhang J. Chen L. Wang J. Butaye P. Huang K. Qiu H. . (2018). Molecular detection of colistin resistance genes (*mcr-1* to *mcr-5*) in human vaginal swabs. BMC. Res. Notes 11:143. doi: 10.1186/s13104-018-3255-3, 29463301 PMC5819219

[ref23] Zhang X. W. Huang X. Y. Zhou Z. Y. Li B. L. Lu J. H. Song J. J. . (2025). Genetic framework and evolutionary dynamics of mcr-positive *Klebsiella pneumoniae* from 2000 to 2023. Int. J. Antimicrob. Agents 66:107533. doi: 10.1016/j.ijantimicag.2025.107533, 40345343

