# Supplementary figures and images for: Prevalence, characteristics, and plasmid dynamics of mcr-1 positive Enterobacteriaceae in Hainan, China: a preliminary genomic investigation
Source: Front Microbiol. 2026 Jan 14;16:1689159. doi: 10.3389/fmicb.2025.1689159 (PMC12848926; doi:10.3389/fmicb.2025.1689159)

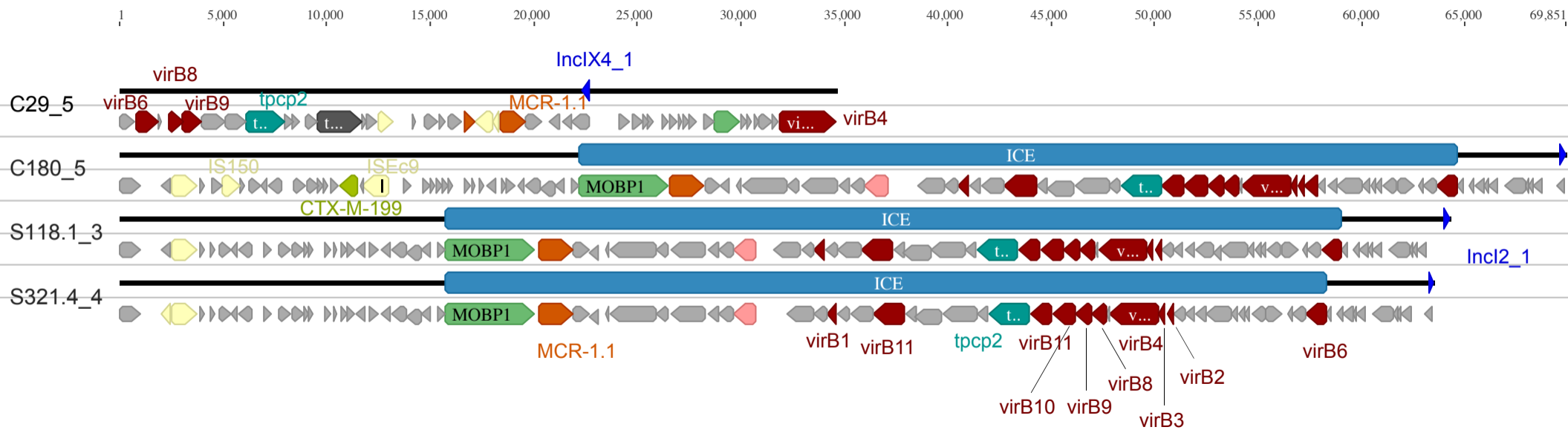

Supplement: Supplementary file 1 [file Data_Sheet_1.PDF]
